# The state of gender diversity in medical physics

**DOI:** 10.1002/mp.14035

**Published:** 2020-02-12

**Authors:** Elizabeth L. Covington, Jean M. Moran, Kelly C. Paradis

**Affiliations:** ^1^ Department of Radiation Oncology University of Alabama‐Birmingham Birmingham AL 35294 USA; ^2^ Department of Radiation Oncology University of Michigan Ann Arbor MI 48109 USA

**Keywords:** diversity, equity, gender

## Abstract

The purpose of this study was to quantify gender diversity in leadership positions within the field of medical physics, as well as within award categories and other recognitions by the American Association of Physicists in Medicine. The April 2019 PDF version of the AAPM membership directory was searched for all users self‐reporting as holding a leadership position at their place of employment, those elected to leadership positions within the AAPM, those serving as chair of an AAPM council, and those listed as having received an award or other such recognition from AAPM (beginning in 1972 with the William D. Coolidge Award). Historical data for these categories were obtained from archived membership directories on the AAPM website. The AAPM website was also used to identify members who have served on the Medical Physics Editorial Board. The Commission on Accreditation of Medical Physics Education Programs (CAMPEP) website was used to identify the current directors of graduate and residency programs (as of July 2019). Because gender was not a reported field in any of these categories, gender was assigned by reviewing names and photographs. Percentage representation in these respects was compared to the overall percentage of women in the AAPM in 2019 (23.3%) and reported the number of women working as medical physicists globally (29.8%). Within the AAPM, the percentage of women reporting clinical leadership roles is 12.0% within the US, 13.6% in Canada, and 18.0% in all other countries combined. Women comprise only 7.5% of CAMPEP graduate program directors and 21.5% of residency program directors. The percentage of female presidents in AAPM is 8.1%. A woman has never served as Editor‐in‐Chief of Medical Physics, and the average for the past 10 yr for female board membership is 13.6%. With the exception of the John R. Cameron Young Investigators Symposium Award, the percentage of all female AAPM awardees is less than the percentage of women AAPM members. The lowest percentage of female representation within AAPM is among council chairs with only one woman having held a chair position out of 42 positions (2.4%) from 1970 to July 2019. Similar to the traditional discipline of physics, medical physics displays a clear gender disparity with regard to leadership positions, both within educational training programs and the AAPM. Further investigation into the demographics of the field and psychosocial factors affecting medical physicists may help to elucidate the origin of these disparities and inform strategies to address them.

## 1 Introduction

The benefits of diversity within higher education and the field of medicine have been widely studied.^1^, ^2^, ^3^, ^4^, ^5^, ^6^ Gurin et al.^2^ demonstrated a positive relationship between interaction with a diverse set of peers and educational outcomes in undergraduate education. Educational benefits of diversity have been documented in medical school for both white and underrepresented minority students: a survey of white medical students who attended programs with the highest quintile for racial and ethnic diversity were “more likely to rate themselves as highly prepared to care for minority populations compared with those from schools in the lowest quintile for diversity.”^4^ Diversity in the physician workforce has a positive social impact since minority and women physicians have been shown to be more likely to serve patients who are racially/ethnically underrepresented, have low socioeconomic status, and/or receive Medicaid.^7^ In the business sphere, even more work has been done to prove the benefits of diversity. For example, a report by management consulting firm McKinsey & Company from January 2018 entitled “Delivering through Diversity” examined over 1000 companies in 12 countries and the benefits of diverse leadership teams. In fact, the most gender‐diverse leadership teams were “21% more likely to outperform on profitability and 27% more likely to have superior value creation.”^8^

Despite such data supporting the many benefits of diversity, many science‐based fields still fall far short of gender parity. One of the most notable gender disparities is within the field of physics. The American Institute of Physics (AIP) has reported extensively on the dearth of women in physics, with recent reports showing that the number of women graduating with a bachelor's degree in physics (21%) has not increased within the past 10 yr.^9^ Of all the STEM fields, physics has the lowest 5‐year average (2013–2017) of the percentage of women doctoral degrees(19%).^10^ In 1988, Dr. Sheila Widnall, former Associate Provost of Massachusetts Institute of Technology and the first women to lead a branch of the military as the Secretary of the United States Air Force, reported on the potential for rapid increases in women participating in science but warned of pipeline leaks where large numbers of women leave the scientific career track at key points in their education.^11^ Thirty years later, these leaks in the pipeline continue to be an issue globally for recruiting and retaining women in science education programs and careers.^12^, ^13^, ^14^, ^15^

There is little published quantitative data on diversity within the field of medical physics. A 2015 report by the International Organization of Medical Physics (IOMP) found that the global percentage of female medical physicists was 28%.^16^ A 2018 study showed that Europe and Latin America had percentages of female medical physicists of 34% and 33%, respectively, while the United States (US) reported only 23% indicating that medical physics gender diversity parallels traditional physics within the USA.^17^ Yet this is not for lack of awareness: more than 10 yr ago in 2008, Dr. Mary K. Martel alerted the AAPM membership to the lack of gender diversity in leadership positions within the AAPM in her Chairwoman of the Board's Column in the AAPM Newsletter.^18^

In this study, we attempt to quantify the gender diversity of leadership roles in medical physics. We quantify the number of women self‐reporting as holding chief or directorship positions, women who serve as the program director of a CAMPEP accredited medical physics graduate or residency program, and the percentage of women serving as editors or on the editorial board of the journal Medical Physics. To further explicate trends in gender diversity, we quantify the number of women who are fellows of AAPM and those who have received an award from AAPM. To our knowledge, this is the first study to report on these gender diversity statistics within the field of medical physics.

## 2 Materials and methods

AAPM Headquarters provided historical data of society membership self‐reported gender breakdown from 1969 to 2019 in 10‐year increments. The April 2019 AAPM directory PDF was downloaded from the AAPM website on July 3rd, 2019; data were parsed via Matlab (MathWorks, Natick, MA) to look for members who self‐identified with the following leadership titles: chief, director, principal, and head, as well to identify those members who were AAPM awardees. Those identified were further stratified by gender and country of practice. Gender was assigned by examining the member’s name and photograph if available. LinkedIn (://www.linkedin.com) profiles and institutional websites were also used to supplement gender assignment, as needed. Sub‐division directors (e.g., director of brachytherapy) and assistant chiefs were excluded from this study because of the lack of consistent and reliable reporting in the directory. We also excluded physicists practicing as consultants or working in government or industry positions.

Historical data regarding AAPM leadership positions were identified by using the archive of directories available on the AAPM website. The AAPM website was also used to identify members who have served on the Medical Physics Editorial Board; members were only included if listed as a direct member and not as a result of serving on another committee. Associate editors were excluded from the analysis. Directors of CAMPEP accredited residency and graduate programs were identified via the CAMPEP website (://www.campep.org). Gender was assigned as described above. Similar to above, assistant directorship positions were excluded from this study. All PDF directories that were used as part of this study were removed from local storage at the conclusion of data collection.

## 3 Results

**Figure 1:**
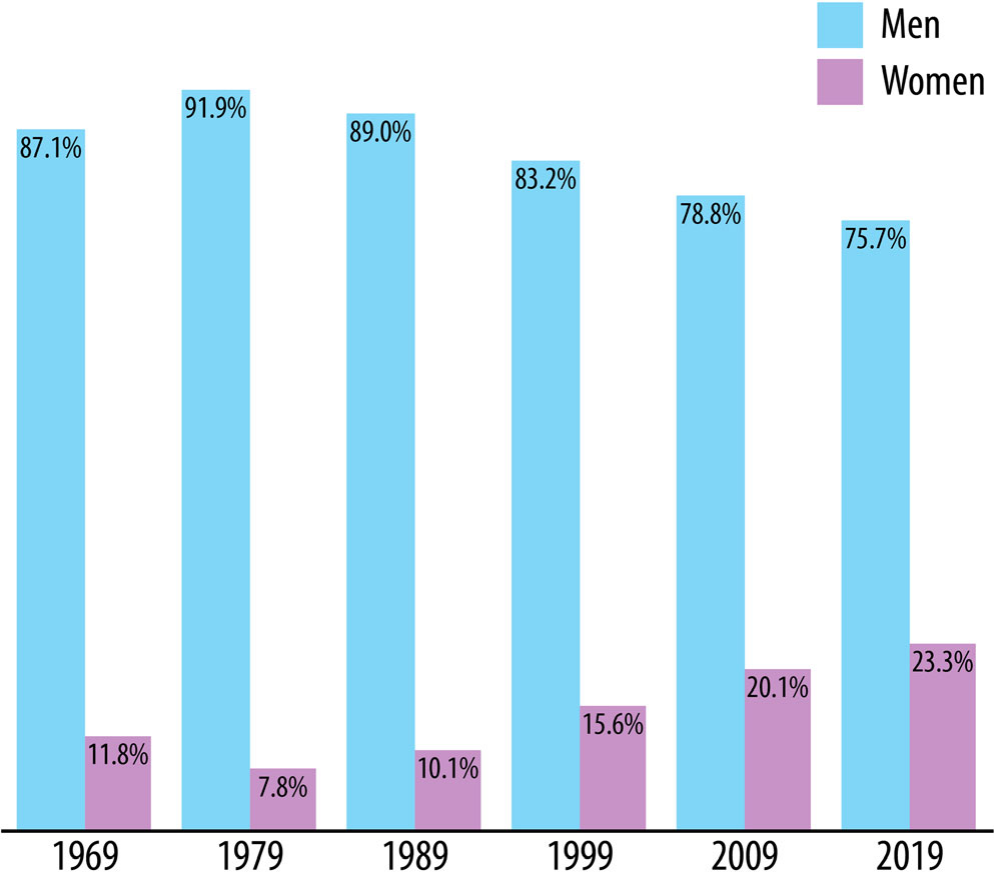
Percentage of male and female AAPM members for the listed year in 10‐yr increments from 1969 to present. [Color figure can be viewed at http://wileyonlinelibrary.com]

Figure 1 shows the distribution of male and female AAPM members for each year in 10‐year increments since 1969 as provided by AAPM using available self‐reported data. Table 1 shows a summary of the remaining data collected for this study.

**Table 1:** Gender distribution among self‐reported clinical leadership positions, CAMPEP program directors, AAPM Council chairs, the AAPM Executive Committee, the Medical Physics Editorial Board, and AAPM awardees. Whether the data are for the current year alone, or including historical data, is noted in each category.

	Total #	Males	Females
Clinical leadership positions (2019)			
USA	524	461 (88%)	63 (12%)
Canada	22	19 (86%)	3 (14%)
Other countries	128	105 (82%)	23 (18%)
TOTAL	674	585 (87%)	89 (13%)
CAMPEP program directors (2019)			
Graduate program	53	49 (93%)	4 (7%)
Residency – imaging	28	22 (79%)	6 (21%)
Residency – therapy	102	80 (78%)	22 (22%)
Residency (all)	130	102 (78%)	28 (22%)
AAPM council chairs (incl. historical)			
Science council	14	14 (100%)	0 (0%)
Education council	13	13 (100%)	0 (0%)
Professional council	13	13 (100%)	0 (0%)
Administrative	2	1 (50%)	1 (50%)
AAPM executive committee (incl. historical)			
President	62	57 (92%)	5 (8%)
Secretary	19	16 (84%)	3 (16%)
Treasurer	16	9 (56%)	7 (44%)
Medical physics editorial board (incl. historical)			
Member	135	118 (87%)	17 (13%)
Editor‐in‐Chief	8	8 (100%)	0 (0.0%)
AAPM awardees (incl. historical)			
Farrington Daniels Award	139	132 (95%)	7 (5%)
William D. Coolidge Award	47	44 (94%)	3 (6%)
Jack Fowler Junior Investigator Award	14	13 (93%)	1 (7%)
Edith H. Quimby Lifetime Achievement Award	44	39 (89%)	5 (11%)
Marvin M.D. Williams Professional Achievement Award	33	29 (88%)	4 (12%)
AAPM Fellowship	570	499 (88%)	71 (12%)
Moses & Sylvia Greenfield Award	147	126 (86%)	21 (14%)
Journal of Applied Clinical Medical Physics Best Paper Award	51	42 (82%)	9 (18%)
Innovation in Medical Physics Education Award	11	9 (82%)	2 (18%)
Science Council Session Winners	138	109 (79%)	29 (21%)
John R. Cameron Young Investigator Symposium Winners	49	35 (71%)	14 (28%)

**Figure 2:**
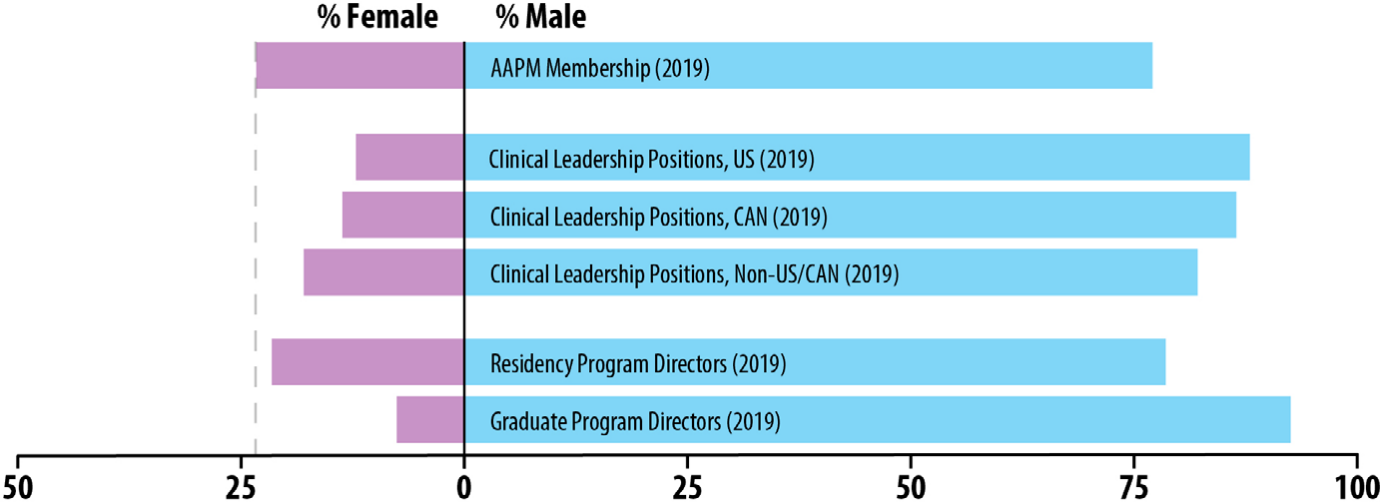
Percentage of males and females identified as clinical leaders in the 2019 AAPM directory, and 2019 residency and graduate program directors, compared to the overall 2019 AAPM membership gender breakdown. [Color figure can be viewed at http://wileyonlinelibrary.com]

**Figure 3:**
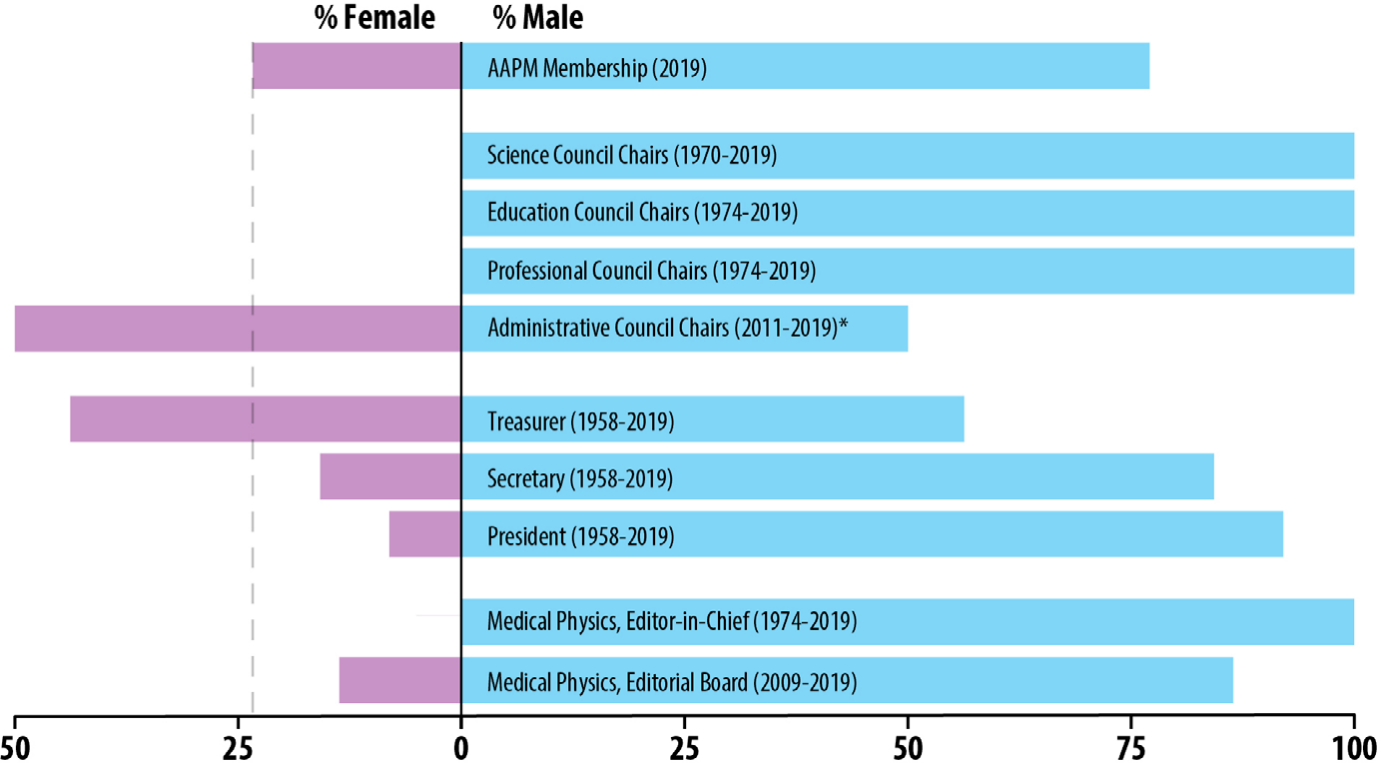
Percentage of male and female AAPM council chairs, members of the Executive Committee, and the Medical Physics editorial board compared to the overall 2019 AAPM membership gender breakdown. *Note that the Administrative Council has had 1 female chair out of 2 total positions since 2011. [Color figure can be viewed at http://wileyonlinelibrary.com]

**Figure 4:**
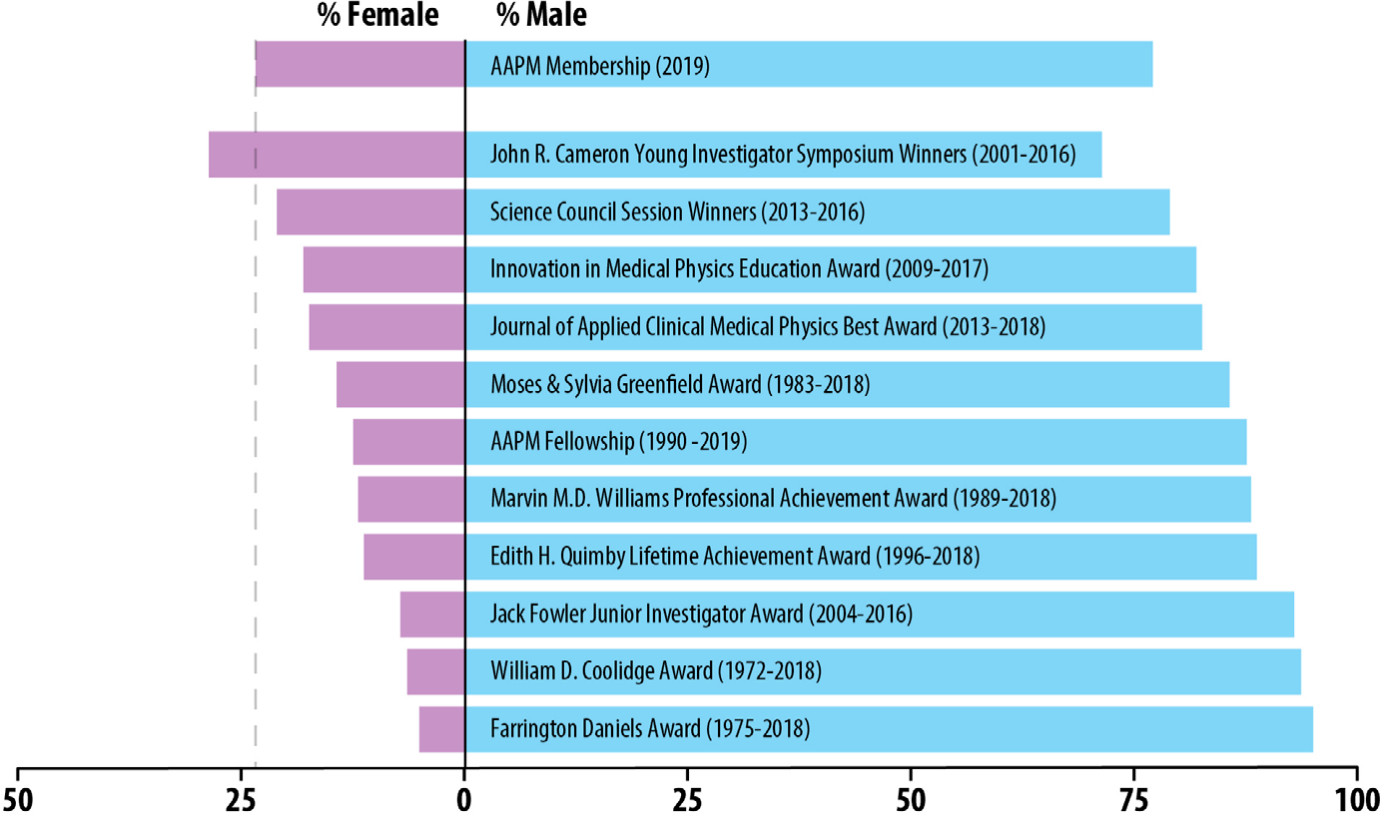
The percentage of male and female AAPM awardees compared to the overall 2019 AAPM membership gender breakdown. [Color figure can be viewed at http://wileyonlinelibrary.com]

The 2019 AAPM directory contained 8939 members, and 841 were identified as holding a leadership position. After the exclusion of sub‐division directors, assistant chiefs, consultants, and physicists working government or industry, 682 leaders remained. Of these 682, the gender of eight members could not be reliably assigned and was omitted from further analysis. Figure 2 shows the percentage of males and females identified as clinical leaders, graduate program directors, and residency directors. Figure 3 shows the percentage of male and female AAPM council chairs, members of the executive committee, and Medical Physics Editorial Board members by year. The percentage of male and female AAPM awardees is shown in Fig. 4. Some of the awardees were excluded from the results if their gender information was not available based on the sources listed above. This happened most often when the winner was not an AAPM member and therefore did not have an entry in the directory; alternatively, some award winners' names were listed with initials only. The excluded awardees included one fellow, seven winners of the Farrington Daniels Award, 28 winners of the Moses & Sylvia Greenfield Award, three winners of the Journal of Applied Clinical Medical Physics Best Paper Award, and 12 Science Council Session Winners. Note that all figures contain the gender breakdown of 2019 AAPM members, with a vertical line representing the percent of female AAPM members (23.3%) for ease of comparison.

## 4 Discussion

In all geographical regions studied, the percentage of women identified as holding a leadership position was lower than the percentage of female AAPM members; in the US, the percentage of female leaders was 12.0%. The group with the lowest percentage of women in leadership positions was CAMPEP‐accredited graduate programs with 7.5%. Three of the four AAPM councils (Science, Professional, and Education) have never had a woman serve as chair. The position in the AAPM executive committee with the highest percentage of women is treasurer at 43.8%, which is more than double the percentage representation as the other positions. This could be attributed to the trend of women volunteering for and being asked to hold positions with lower upward mobility^19^ rather than positions that are “escalators” to leadership positions.^20^ While Fig. 1 reports 50% of positions in Administrative council were female, there has been only one woman who served as chair from 2011 to 2015 out of two total chair positions since its inception in 2011; therefore, the percentage of female AAPM Council chairs since 1970 is 2.4% (1 of 42). A woman has never held the position as Editor‐in‐Chief of the journal Medical Physics.

Gender disparities in scientific awards have shown that not only do women receive fewer awards, but they also receive less money and prestige.^21^ The AAPM award category with the lowest percentage of women awardees is the Farrington Daniels Award, which is for an outstanding paper on radiation therapy dosimetry, planning or delivery published in the journal Medical Physics in the previous calendar year. This is concordant with other studies that have shown a gender disparity in the number of women who receive research funding^22^ and participate in academic activities.^23^ Gender‐blind assessment has been shown to be an effective strategy to overcome disparities in funding. The Irish Research Council introduced gender‐blind assessment in 2014, and the percentage of women receiving funding increased from 35% in 2013 to 57% in 2017.^24^ Within the AAPM, the award category with the highest percentage of female awardees is the John R. Cameron Young Investigator Symposium.

While physicist diversity in the field of radiation oncology has not been previously reported on, diversity among radiation oncologists has been studied and may offer insight regarding barriers for equity.^3^, ^20^, ^25^, ^26^, ^27^, ^28^, ^29^, ^30^, ^31^ A 30‐yr analysis of gender trends in radiation oncology by Ahmed et al. shows that although the percentage of female faculty members has been increasing, women remain underrepresented in the field.^25^ According to a 2017 report, 27.7% of radiation oncology faculty were women.^26^ Knoll et al.^20^ directly addressed the issue of gender leadership disparity in radiation oncology. Similar to this study, they showed that the number of women on the American Society for Radiation Oncology (ASTRO) board of directors and Gold Medal awardees did not reflect the percentage of women in the field. Women have only been 2 of 46 (4.3%) ASTRO presidents and 9 of 82 (11.0%) of academic radiation oncology chairs as of 2018. Knoll et al. theorize that the dependence of networking with male colleagues may be a barrier for women’s progress and/or women may be intentionally excluded from senior leadership positions.

We note that there are limitations to this work. For the purpose of this study, gender assignment was a binary variable determined by the authors from the perceived gender of the name along with consideration of photographs from either the AAPM directory or another publically available website. We did not have self‐reported gender data with which to base our analysis and we acknowledge that gender is not binary.^32^ Historical data provided by AAPM Headquarters was also compiled using available self‐reported gender as reported in 2002 to determine the gender of members prior to 2009.

This study also assumed that self‐reported leadership status in the AAPM directory was accurate; leadership status was not verified for each member. We acknowledge that the data may be out of date and self‐reported data may contain inaccuracies. The analysis was limited to those reporting leadership in 2018 and therefore we do not have a history of prior leadership positions for any individuals. Since this is the first study to quantify leadership positions with the medical physics community, we believe that the data in its current state is nonetheless valuable to members of our community, especially with respect to identifying failures to equitably support and promote all members of AAPM to higher ranks in the field.

While statistics concerning gender diversity are important, they is only one piece of the larger puzzle of diversity, equity, and inclusion in the field of medical physics. It is not possible to draw concrete conclusions regarding the reasons for the afore‐described disparities given only these statistics. There is, therefore, a need to allow AAPM members to self‐report race, ethnicity, and gender in ways that accurately represent their identities, as well as to solicit confidential information from the membership regarding psychosocial factors that result in asymmetric representation, leadership, and/or success in the field. Given that one of the strategic goals of the AAPM, added in 2018, is to “champion equity, diversity, and inclusion (EDI),” both of these new initiatives would complement the aims of our professional society and support the vitality of our organization as a whole.

Equity, diversity, and inclusion initiatives can be controversial, particularly as they relate to the best strategies to approach and address the current state of diversity within our field. First, we must understand the current climate and roadblocks as perceived by our members to begin to address the disparities. Through surveys and outreach programs, we can begin to uncover the barriers and develop appropriate interventions to strengthen our field as a whole. Without a concerted effort by AAPM and the medical physics community at large, the state of gender diversity in medical physics is on track to remain imbalanced for the next half‐century: if the number of female AAPM members continues to increase at the current pace of 0.4% per year, it will take *nearly 70 yr* to achieve gender parity. To overcome this slow pace, our organization must not only declare EDI a strategic goal (which it has done), but also implement policies and initiatives to accelerate progress toward overcoming the barriers to equity. Holliday et al.^33^ discuss the barriers to achieving gender equity in the physician radiation oncology workforce and highlight the importance of unconscious bias training. Unconscious bias training could be offered as a yearly self‐assessment module session at the annual meeting, and department leaders should consider annual training modules on unconscious bias in addition to regulatory training. AAPM and its associated peer‐reviewed journals should consider gender blind review of all awards, proposals, and publications. Salary inequity could also be better examined in the annual AAPM Professional Survey to more explicitly compare across genders while controlling for other factors such as years of service. AAPM‐sponsored mentorship programs to match students and junior members of the organization with established women and men in the field could help address leaky pipeline issues and build a support network. AAPM should also vocally support parental leave policies and consider financial support for students and trainees who are not covered by such policies to address another source of the leaky pipeline. Educational programs that are targeted to addressing inequities may ultimately be beneficial for both women and men.

While this work has focused on gender diversity, we believe that there is important work to be done on all aspects of EDI within the AAPM and in medical physics. The AAPM currently has two committees with a focus on EDI: the Diversity and Inclusion Subcommittee and the Women’s Professional Subcommittee. To remedy gender inequity within AAPM, cross‐council efforts will be needed which span the organization as a whole.

## 5 Conclusions

Gender representation in leadership positions in medical physics does not reflect the percentage of women working in the field. With the exceptions of the AAPM treasurer positon and the John R. Cameron Young Investigator Symposium winners, the percentage of female medical physicists in leadership positions, AAPM fellows, executive committee members, Medical Physics board members, council chairs, and AAPM awardees, all are below the percentage of female medical physicists globally. While residency director positions are relatively well‐aligned with the percentage of female medical physicists, clinical director positions and graduate program directors are much lower. This analysis is beneficial for those seeking champion diversity within both the AAPM and the international medical physics community.

## Conflict of interest

The authors have no conflict to disclose.
